# Commentary: Why Are No Animal Communication Systems Simple Languages?

**DOI:** 10.3389/fpsyg.2021.722685

**Published:** 2021-10-04

**Authors:** Dustin J. Penn, Szabolcs Számadó

**Affiliations:** ^1^Department of Interdisciplinary Life Sciences, Konrad Lorenz Institute of Ethology, University of Veterinary Medicine, Vienna, Austria; ^2^Department of Sociology and Communication, Budapest University of Technology and Economics, Budapest, Hungary; ^3^Center for Social Sciences, Eötvös Loránd Research Network (ELKH), Budapest, Hungary

**Keywords:** language, human evolution, animal communication, handicap principle, gene-culture co-evolution

“Language, too, has apparently evolved only in us: that is to say 40 times less often than eyes. It is surprisingly hard to think of 'good ideas' that have evolved only once.”–Dawkins (2004), p. 592

Ever since Darwin, scientists have sought to understand the origins of human language, which has been called “the hardest problem in science” (see Christiansen and Kirby, 2003; Számadó and Szathmáry, 2006 for review). It is difficult to understand why humans are the only species that evolved language. Beecher (2021), a pioneer in the study of birdsong, recently considered this problem and the implications of animal communication research for the evolution of language. He did a splendid job describing the “design features” of language vs. other animal communication systems. However, his summary of honest signaling theory is inaccurate, and he overlooked gene-culture co-evolution for explaining language.

Beecher first clarifies the design features that make human language different from the communication systems of other species (i.e., semanticity, nearly infinite information capacity, arbitrariness, evolvability via cultural transmission). He then provides a clear explanation for why the communication of other species lacks the key features of a full-fledged language, and examines birdsong as an example. Song birds have vocal learning, a complex vocal repertoire, and hierarchical structuring of vocal signals, but they do not seem to use these capacities to form different songs to represent different meanings (birds do not transmit different messages with different songs; they do not seem to create new meanings by recombining words into sentences). Birdsong has complexity, but not infinite information capacity. He convincingly argues that animal vocalizations do not function like language.

He then examines the debates over the “fundamental nature of animal communication,” and whether animal signals are honest or manipulative; but this summary is not completely accurate. Dawkins and Krebs (1978) challenged the widespread assumption that animal signals are always honest and function to provide information *per se*, and pointed out that their function is to influence conspecifics, which can include persuasion, deception, and manipulation. Some have mistakenly pitted information against influence (Rendall et al., 2009), which are not alternatives; signals can be influential because they inform—or misinform. The main issue at stake here is whether we should expect signals to be honest, but contrary to what Beecher assumes, there is no theoretical justification for the idea that they should be honest *on average*. Animals should evolve sales resistance to avoid manipulation, as he points out, but there are many examples of deception, and the amount deception can be high in theoretical models.

Beecher argues that the handicap principle will maintain some degree of honesty in any signaling system, even though it is “still being subjected to further modification and clarification” (p. 10), and cites our recent review (i.e., Penn and Számadó, 2020). However, we argue that the handicap principle needs to be fully rejected, not modified. The handicap principle is the idea that signals are honest because they are costly to produce, and it assumes that signals are costly is to demonstrate that they are honest. Its logic is circular and non-Darwinian (costly traits evolve despite and not because of their costs), and since theoretical models have shown that signaling costs are not not necessary to maintain honesty (see below), this idea can and should be rejected.

Beecher states that the handicap principle was formalized by Grafen's (1990) model, but this is not a handicap model and it never was. Grafen never showed that signals will be wasteful at the equilibrium, and his equations do not support his handicap interpretation, as they show the necessity of differential marginal costs, and not the necessity of equilibrium costs for honest signaling. Yet, it is not the equilibrium cost of signals (a.k.a. handicaps) that maintain honesty, but the potential cost of cheating (i.e., ratio of marginal benefit to marginal cost for potential cheaters, see Hurd, 1995; Lachmann et al., 2001; Bergstrom et al., 2002). Neither the handicap principle nor Grafen's “main handicap results” are supported by theoretical models or empirical results (see Számadó, 2011; Penn and Számadó, 2020). Beecher suggests that some studies on peacocks provide “a clear illustration of the predictions generated by the handicap principle, and how they should be tested.” He is actually referring to Grafen's model rather than the handicap principle, but neither were tested in these studies. Also, Grafen's model is not as general as Beecher assumes, and it is unclear that it can explain signal reliability (e.g., see Nöldeke and Samuelson, 2003).

The handicap principle has generated enormous confusion due to its dressing up a non-Darwinian idea (handicaps) as a Darwinian theory and scientific principle. It misled a generation of biologists into attempting to measure the equilibrium cost of signals, which is uninformative, instead of measuring the marginal costs and marginal *fitness* benefits of signals. Animal communication can be described and analyzed in terms of evolutionary life-history trade-offs, without imposing the confusing language of the handicap paradigm.

Finally, Beecher argues that the key ingredient for the evolution of language is zero conflict of interest between sender and receiver. We humans are exceptional for our capacity to cooperate with strangers (Maynard Smith and Szathmáry, 1995); but we are far from zero conflict. Our species' success seems to be due to our ability to cooperate in large numbers toward common goals, which undoubtedly requires language. It is unclear, however, whether cooperation drove the evolution of language or vice versa. It is difficult to see how one might determine which came first (but see Számadó, 2010), and they likely co-evolved. Darwin (1871) proposed that languages evolve like living organisms, and gene-culture co-evolutionary theory provides important insights into how language and cooperation can influence each other's evolution (e.g., Pinker and Bloom, 1990; Richerson and Boyd, 1999; Számadó and Szathmáry, 2012; see concise summary in Richerson et al., 2021). As language and cooperation evolved, they may have generated positive feedback with each other (e.g., Számadó, 2010). Language likely allowed our ancestors to cooperate, and helped to resolve conflicts by exchanging information, though this includes invented fictions, social constructions, and other imagined realities (Harari, 2014). Honest communication is corruptible, as long as there are conflicts of interest (Dawkins and Guilford, 1991). Yet, for language to function as it does, it need not be completely reliable (and clearly it is not) and conflicts need not be zero; it only needs to facilitate communication.

## Author Contributions

DP and SS wrote the commentary. All authors contributed to the article and approved the submitted version.

## Funding

DP acknowledges support from the Austrian Science Fund (AT): P28141-B25 of the Austrian Science Foundation. SS was supported by OTKA grant K132250, and by the European Structural Investment Funds (ESIF) in Hungary (GINOP) 2.3.2-15-2016-00057. The funders had no role in study design, data collection and analysis, decision to publish, or preparation of the manuscript.

## Conflict of Interest

The authors declare that the research was conducted in the absence of any commercial or financial relationships that could be construed as a potential conflict of interest.

## Publisher's Note

All claims expressed in this article are solely those of the authors and do not necessarily represent those of their affiliated organizations, or those of the publisher, the editors and the reviewers. Any product that may be evaluated in this article, or claim that may be made by its manufacturer, is not guaranteed or endorsed by the publisher.
